# A meta-analysis of the efficacy and safety of adjuvant sorafenib for hepatocellular carcinoma after resection

**DOI:** 10.1186/s12957-021-02280-9

**Published:** 2021-06-10

**Authors:** Shenglan Huang, Dan Li, LingLing Zhuang, Liying Sun, Jianbing Wu

**Affiliations:** 1 Department of Oncology, The Second Affiliated Hospital of Nanchang University, No. 1, Minde Road, Nanchang, 330006 Jiangxi Province People’s Republic of China; 2 Department of Gynaecology, The Second Affiliated Hospital of Nanchang University, No. 1, Minde Road, Nanchang, 330006 Jiangxi Province People’s Republic of China

**Keywords:** Hepatocellular carcinoma, HCC, Sorafenib, Resection, Meta-analysis

## Abstract

**Background:**

Sorafenib was reported as a useful adjuvant treatment in patients with hepatocellular carcinoma who underwent surgical resection. However, its therapeutic value remains controversial. This meta-analysis examined the available data regarding the efficacy and safety of sorafenib in patients with hepatocellular carcinoma after radical surgery.

**Methods:**

The meta-analysis was conducted according to the Preferred Reporting Items for Systematic Reviews and Meta-Analyses guidelines. The protocol was registered in advance with PROSPERO (CRD42021233868). We searched PubMed, Embase, Cochrane Library, and Web of Science to identify eligible studies. Overall survival, recurrence-free survival, and recurrence rates were analyzed, and adverse events were reviewed. Hazard ratios or pooled risk ratios with 95% CIs were collected and analyzed using STATA version 12.0 in a fixed-effects or random-effects meta-analysis model.

**Results:**

In total, 2655 patients from 13 studies were ultimately included in this meta-analysis. The combined results illustrated that sorafenib was associated with better overall survival than the control (hazard ratio = 0.71, 95% CI = 0.59–0.86; *P* < 0.001). Similarly, the drug also improved recurrence-free survival (hazard ratio = 0.68, 95% CI = 0.54–0.86, *P* = 0.001). Combined data revealed that patients treated with sorafenib after resection had a lower recurrence rate (pooled risk ratio = 0.78, 95% CI = 0.68–0.90, *P* < 0.001). The primary adverse events were hand-foot skin reaction, fatigue, and diarrhea of mild-to-moderate severity, whereas grade 4 adverse events were rare (< 1%).

**Conclusions:**

This meta-analysis demonstrated that adjuvant sorafenib therapy after resection in patients with hepatocellular carcinoma could prolong overall survival and recurrence-free survival and reduce recurrence rates without intolerable side effects. However, more evidence is needed before reaching a definitive conclusion.

## Background

Liver cancer is currently the fourth common cause of cancer-related mortality and the sixth most common cause of cancer, and the World Health Organization predicts that more than one million patients will die from liver cancer in 2030 [1, 2]. The prevalence of hepatocellular carcinoma (HCC), which accounts for 75–80% of liver cancers, has increased in recent years [3]. Approximately 700,000 new cases of HCC are detected annually, with more than half occurring in developing countries. In addition, 75% of HCC-related deaths occur in Asian countries [4].

At an early stage of HCC (single nodule ≤ 5 cm in diameter or 2–3 nodules ≤ 3 cm in diameter), potentially curative treatments, including surgical resection, radiofrequency ablation (RFA), and liver transplantation, can be applied [5]. In theory, the best treatment option is liver transplantation, but the scarcity of liver donors limits its applicability; hence, most patients undergo resection or RFA as the first-line treatment. Prior research found that 5-year overall survival (OS) rates for the RFA and resection groups were 54.78% and 75.65%, respectively. Regarding solitary huge HCC, a previous study demonstrated that portal vein ligation combined with staged hepatectomy provided superior outcomes to transarterial chemoembolization (TACE) [6]. Even very elderly HCC patients (aged 80 years or older) could potentially benefit from hepatectomy if patients have a good general status, and the prognoses of very elderly patients were similar to nonelderly individuals [7]. Although resection may provide better survival and lower recurrence rates for patients with HCC [8], the recurrence rate after surgical resection remains as high as 50% after 3 years and 70% at 5 years [9]. Factors related to the postoperative recurrence of HCC include the histological tumor grade, presence of vascular invasion, presence of microsatellite nodules, tumor size, and resection margin [10]. Thus, the long-term prognosis after surgery remains unsatisfactory, and systemic adjuvant therapy plays an important role in the treatment of HCC after resection.

Currently, there is no standard adjuvant therapy for HCC after resection. On the basis of its mechanism of inhibiting tumor cell proliferation and angiogenesis, sorafenib has been widely used after gaining approval from the US Food and Drug Administration for the treatment of HCC. Moreover, it is the only approved treatment recommended by the American Association for the Study of Liver Diseases for patients with advanced and unresectable HCC. However, whether sorafenib can suppress postoperative recurrence and consequently prolong survival remains controversial. At present, several retrospective studies have found that sorafenib can reduce the postoperative recurrence rate and improve long-term survival rates in patients with HCC [11–14]. A case-control study revealed that adjuvant sorafenib could prolong disease-free survival and OS in patients with HCC beyond the Milan criteria after orthotopic liver transplantation [15]. Conversely, the STORM trial indicated that sorafenib is not an effective intervention in the adjuvant setting for HCC following resection or RFA [16]. To resolve this controversy, we undertook this meta-analysis to investigate whether adjuvant sorafenib is useful in patients with HCC after resection.

## Methods

### Literature search

This meta-analysis was conducted in accordance with the Preferred Reporting Items for Systematic Reviews and Meta-Analyses guidelines. We comprehensively searched PubMed, Embase, Cochrane Library, and Web of Science using MeSH terms and keywords (liver neoplasm, hepatectomy, sorafenib) to identify relevant clinical trials performed before January 2021. The publication language was limited to English. First, the title and abstract were filtered, and then the reference lists of the retrieved articles were analyzed. The protocol was registered with PROSPERO in advance (CRD42021233868).

### Inclusion and exclusion criteria

Studies eligible for inclusion in this meta-analysis had to meet all of the following criteria: (1) patients with resectable HCC who only underwent surgical resection; (2) the intervention was sorafenib therapy after resection versus observation or placebo before tumor progression or recurrence; (3) the study design was limited to randomized control trials (RCTs), retrospective or prospective cohort studies, and case-control studies; and (4) outcome measures, including OS, recurrence-free survival (RFS), and/or the recurrence rate, were available or could be calculated.

The exclusion criteria were as follows: (1) reviews, abstract, case reports, and letters; (2) the absence of sufficient data to estimate hazard ratios (HRs) and 95% CIs; (3) publication in languages other than English; and (4) the inclusion of duplicate data or repeat analyses.

### Data extraction and quality assessment

After preliminarily evaluating the relevant articles retrieved from the databases mentioned above, two reviewers (Shenglan Huang and Dan Li) screened and extracted relevant articles independently by reading the titles and abstracts. The number of studies in each screening procedure and the reasons for exclusion were recorded. The reviewers then carefully read the full texts of the included studies and extracted useful information. The following information was collected: first author, publication year, country or region, study design, sample size, patient demographics, tumor characteristics, follow-up duration, treatment duration, outcome measures (OS, RFS, recurrence rate) with HRs/risk ratios (RRs) and 95% CIs, adverse events, and type of survival analysis (univariate or multivariate). If both univariate and multivariate analyses were performed in the study, the HRs of multivariate analysis were preferred. Two independent authors (Shenglan Huang and Dan Li) used the Newcastle-Ottawa Scale (NOS) to evaluate the quality of case-control or cohort studies. The Jadad scoring system was applied for RCTs. Jianbing Wu resolved any lack of clarity or disagreement.

### Statistical analysis

HRs and 95% CIs were obtained directly from the articles or calculated from the Kaplan-Meier curves using Engauge Digitizer version 4.1 (http://digitizer.sourceforge. net/). Pooled HRs and their 95% CIs were estimated for OS and RFS. HR < 1 indicated that sorafenib was associated with a better prognosis in patients with HCC after resection. Pooled RRs with 95% CIs were estimated for recurrence rates, and RR < 1 indicated favorable outcomes in the sorafenib group. Cochran’s Q test and Higgins *I*^2^ statistic were applied to assess the heterogeneity of the included studies. Significant heterogeneity among the studies was indicated by *P* < 0.05 or *I*^2^ > 50%, and the random-effects model was adopted to calculate the pooled HRs. Otherwise, the fixed-effects model was used. Subgroup and sensitivity analyses were performed to explore the heterogeneity among the results of different studies. Begg’s test and Egger’s linear regression analysis were conducted to evaluate publication bias. For all outcomes, *P* < 0.05 denoted statistical significance. All statistical analyses were conducted using STATA version 12.0 (STATA Corporation, College Station, TX, USA).

## Results

### Study selection and quality assessment

**Fig. 1:**
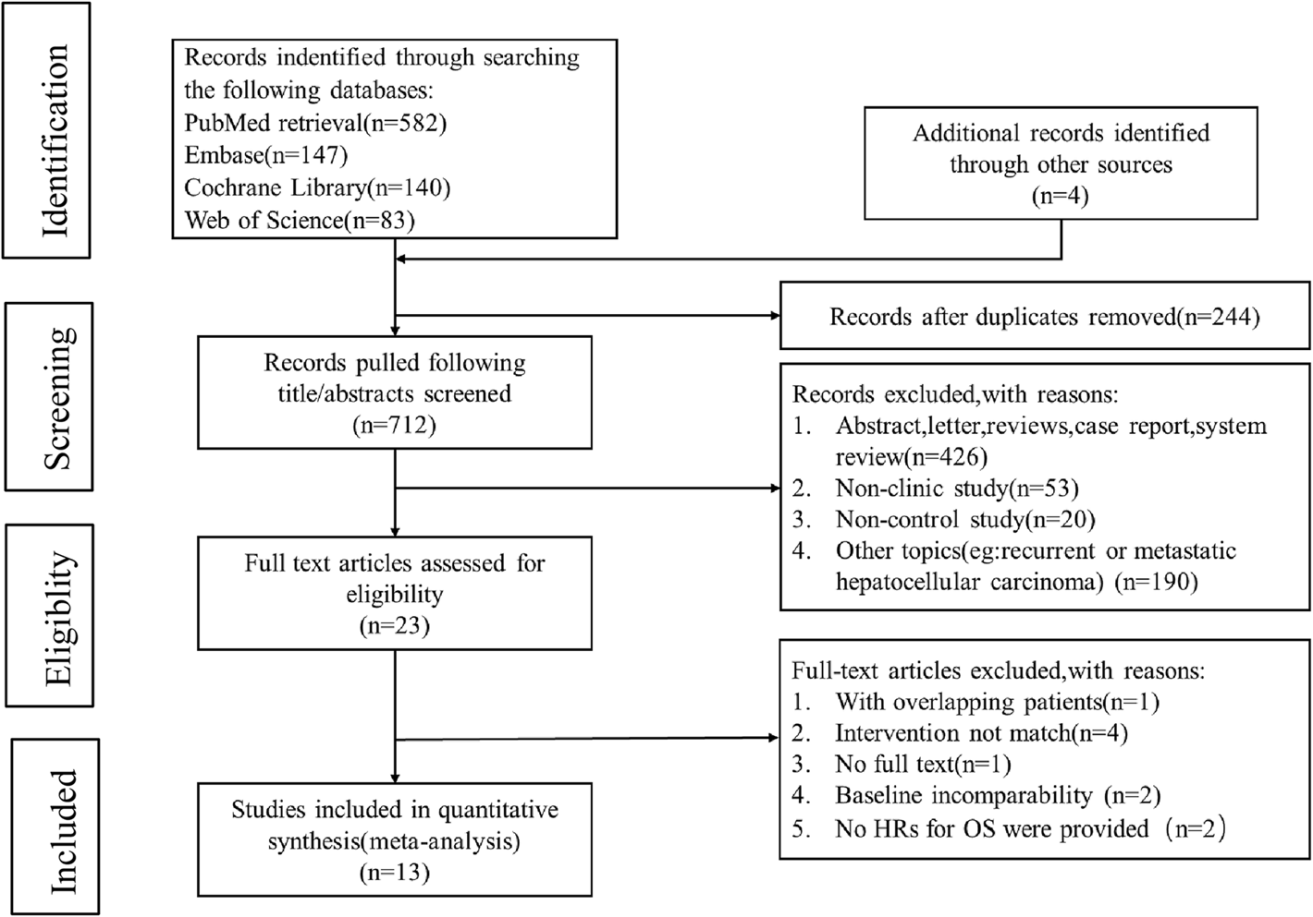
Flow chart of the study selection

In total, 956 records were retrieved through the preliminary search strategies, including 582 records from PubMed, 147 from Embase, 140 from Cochrane Library, 83 from Web of Science, and 4 from other databases. After removing the duplicates, 712 articles were further screened. By reading the titles and abstracts, 679 irrelevant studies were excluded, and the remaining 23 articles were carefully checked according to the inclusion and exclusion criteria. Ten articles that did not meet the inclusion criteria were excluded. Finally, 13 comparative studies involving 2655 patients were included in the meta-analysis [11–14, 16–24]. The screening process of the study is presented in Fig. 1.

Among the 13 studies included in the final analysis, 11 studies were conducted in China; one study was undertaken across 202 sites (hospitals and research centers) in 28 countries throughout the Americas, the Asia-Pacific region, and Europe; and one study included Caucasians. The 13 studies included 10 retrospective studies and 3 prospective studies. A total of 1039 patients in the studies received sorafenib. The eligible studies were all published from 2014 to 2020. The characteristics of each study are listed in Table 1.

**Table 1:** Main characteristics of the studies included in the meta-analysis

Author	Year	Region	Study period	Study design	No. of patients		Age (years; median and range)		Male/female		Follow-up (months; median and range)		NOS or Jadad score
					Sorafenib	Control	Sorafenib	Control	Sorafenib	Control	Sorafenib	Control	
Wang SN	2014	China	2010.5–2012.11	Prospective	14	17	61.43 ± 10.15	59.71 ± 11.34	13/1	15/2	19 (14)	19 (14)	8
Zhang W	2014	China	2009.8–2011.11	Retrospective	32	46	54.5 ± 1.6	51.7 ± 1.4	25/7	42/4	–	–	7
Bruix J	2015	28 countries	2008.8–2010.11	RCT	556	558	58 (24–85)	60 (19–80)	451/105	461/97	23·0 (12.7–36)	22 (14.4–35.5)	7
Antonious	2016	Caucasian	2005.1–2013.1	Retrospective	16	14	62 (55–67.5)	65.5 (53–71)	13/3	10/4	Median 38.2	Median 38.2	7
Li J	2016	China	2009.1–2013.12	Retrospective	12	24	49.8 ± 6	52.8 ± 6.9	12/0	24/0	23 (9–54)	23 (9–54)	7
Xia F	2016	China	2010.9–2013.9	Retrospective case control	34	68	48 (21–78)	57 (18–79)	25/9	50/18	Median 26	Median 25	6
Chen BF	2016	China	2009.6–2012.6	Prospective	24	46	48 ± 10	48 ± 11	22/2	40/6	36–60	36–60	7
Liao Y	2017	China	2010.7–2013.7	Retrospective	14	28	47.4 ± 10.6	48.4 ± 11.0	11/3	26/2	13.6 (1.5–40.1)	13.6 (1.5–40.1)	8
Zhuang L	2017	China	2010.1–2012.7	Retrospective Case control	27	54	48.2 ± 9.7	49.4 ± 9.4	25/2	50/4	14.5 (2.6–44.7)	14.5 (2.6–44.7)	8
Huang Y	2019	China	2009.1–2016.12	Retrospective	16	33	52.25 ± 11.94	51.25 ± 11.87	12/4	30/3	–	–	8
Wang DS	2019	China	2012.1–2013.11	Retrospective	98	111	46.5 (15–77)	55 (30–79)	86/12	100/11	Median 14	Median 15.4	8
Zhang XP	2019	China	2009–2016	Retrospective	113	113	49 (43–56)	48.0 (40–57)	97/16	98/15	–	–	8
Sheng PC	2020	China	2015.5–2018.1	Retrospective	49	36	–	–	36/13	28/8	35.4 (2–40.8)	35.4 (2–40.8)	8

*NOS* Newcastle-Ottawa Scale, *RCT* randomized controlled trial

Regarding sorafenib treatment, 12 of the included studies used an initial dose of 400 mg twice daily, and doses were adjusted according to the safety findings and patient tolerability [11–14, 16–24]. Tumor size, which differed among the studies, was recorded in all included studies. Treatment duration was described in seven studies, ranging from 4 to 70.97 months [12–14, 16, 19, 20, 23]. The characteristics of the sorafenib treatment group are listed in Table 2.

**Table 2:** Main characteristics of patients treated with sorafenib in the included studies

Author	Year	Sample size (sorafenib)	Media age	Male (%)	Tumor size (cm)	HRs	Initial dose (sorafenib)	Duration (months; median and range)	Risk factors
Wang SN	2014	14	61.43	92.9	Median 6.26 ± 2.12	UV/MV	400 mg	4	MVI (78.16%)
Zhang W	2014	32	51.7	78.1	Median 5.7 ± 0.6	UV/MV	400 mg	–	Multiple tumors (53.1%) Portal vein thrombosis (25%) TNM stage III (31.2%)
Bruix J	2015	556	58	81	Median 3.5 (1.0–20)	UV	400 mg	12.5 (2.6–35.8)	MVI (68%)
Antonious	2016	16	65.5	81.3	Median 7.8 (6.0–9.8)	UV	200–400 mg	–	MVI (61.5%)
Li J	2016	12	49.8	100	Largest 9.8 ± 2.1	UV	400 mg	–	BCLC C (100%) portal vein Thrombus (100%)
Xia F	2016	34	48	73.5	Media 6.4 (2.8–20.2)	MV	400 mg	22.9	BCLC C (100%)
Chen BF	2016	24	48	91.67	Media 4.4 (0.8–10.5)	UV	400 mg	6	–
Liao Y	2017	14	47.4	78.6	<10 (71.4%) ≥ 10 (28.6%)	UV/MV	400 mg	14.3 (2.6–24.2)	Tumor size
Zhuang L	2017	27	48.2	92.6%	Media 7.8 ± 3.9	UV/MV	400 mg	7.3 (5.8–8.9)	BCLC B/C
Huang Y	2019	16	52.25	75%	≤5 (37.5%) > 5 (62.5%)	UV/MV	400 mg	45.52 (1.10–70.97)	MVI
Wang DS	2019	98	46.5	87.76	Largest 7.91 ± 3.42	UV	400 mg	–	BCLC B/C
Zhang XP	2019	147	47	87.8	Media 6.0 (4.0–9.6)	UV	400 mg	–	MVI
Sheng PC	2020	49	–	73.4	≤5 (58.3%) > 5 (41.7%)	UV/MV	400 mg	–	MVI

*HR* hazard ratio

The NOS was applied in 12 studies. The NOS was ≥ 6 in all studies, indicating that the included studies had mild-to-moderate risks of bias. The Jadad score was applied in the remaining one RCT, and the score was 7. The results of the quality assessment in the included studies are presented in Table 1.

### Treatment outcomes

#### Overall survival

**Fig. 2:**
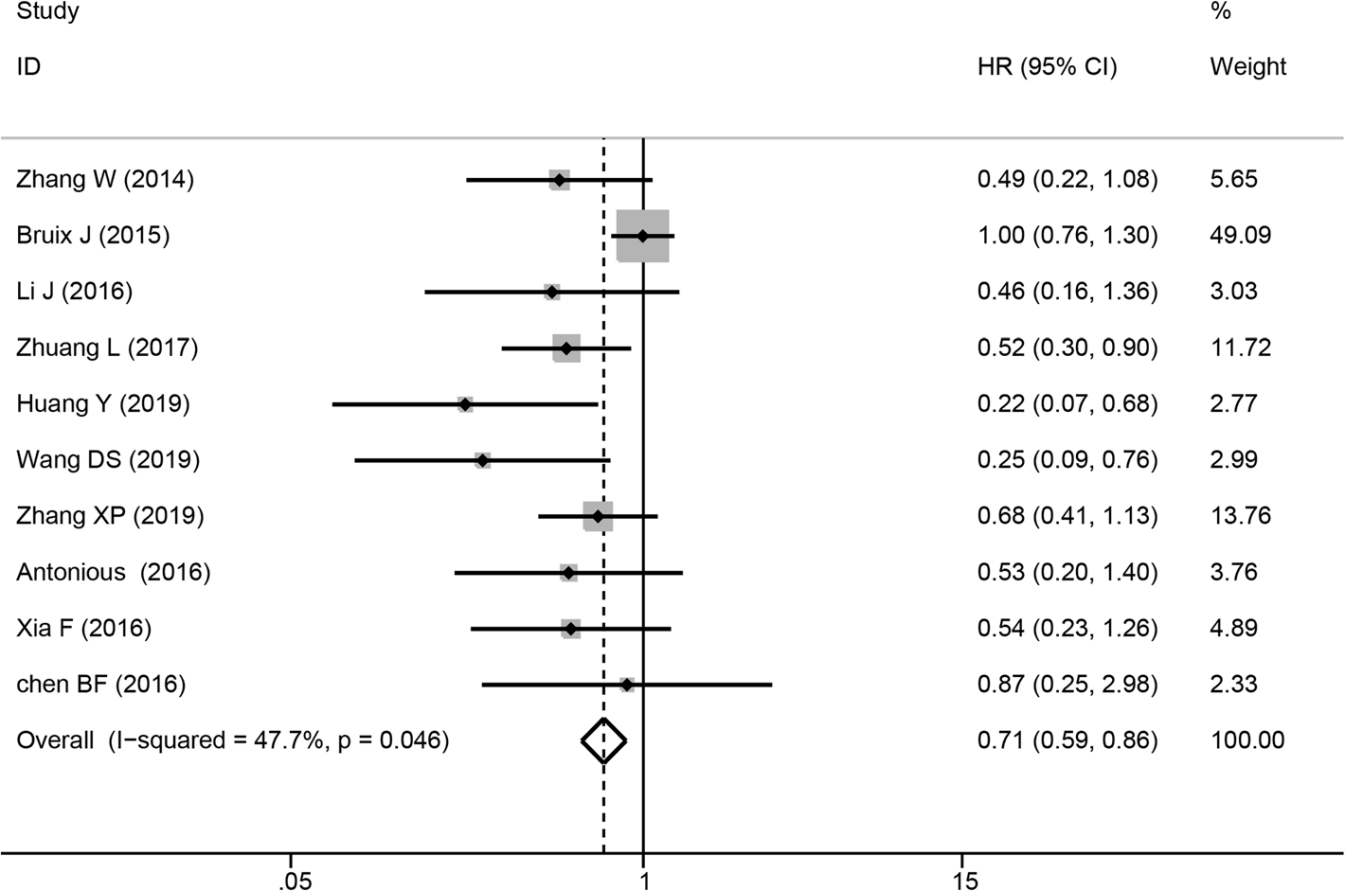
Forest plot of OS outcomes in patients who received sorafenib therapy for HCC after resection. OS, overall survival; HCC, hepatocellular carcinoma

Ten studies involving 1995 patients presented data for OS. Among them, four studies provided multivariate-adjusted HRs (Table 2). The pooled results revealed that sorafenib treatment led to better OS than the control after resection (HR = 0.71, 95% CI = 0.59–0.86, *P* < 0.001), and no significant heterogeneity was observed among the studies (*I*^2^ = 47.7%, *P* = 0.046), as presented in Fig. 2.

Begg’s funnel plot and Egger’s test were used to evaluate publication bias. Publication bias for OS was detected (Pr > |z| = 0.283 for Begg’s test and P > |t| = 0.003 for Egger’s test). The corrected pooled HR for OS was not changed after using the “trim and fill” method to adjust for publication bias (HR = 0.71, 95% CI = 0.59–0.86, *P* < 0.001).

#### Recurrence-free survival

**Fig. 3:**
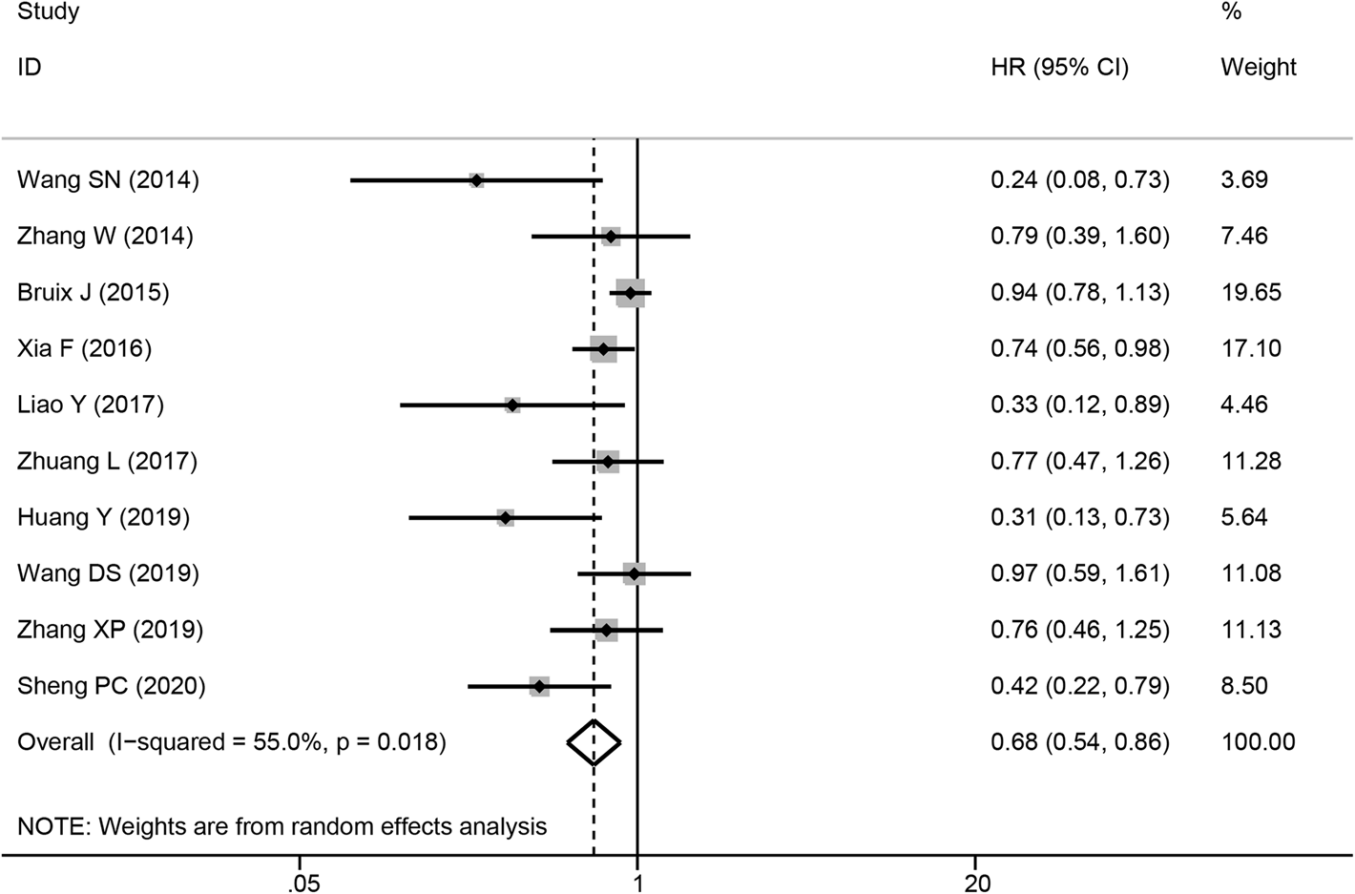
Forest plot of RFS outcomes in patients who received sorafenib therapy for HCC after resection. RFS, recurrence-free survival; HCC, hepatocellular carcinoma

Ten trials reported a correlation between sorafenib treatment and RFS. The forest plot indicated that the HR for RFS was 0.68 (95% CI = 0.54–0.86, *P* = 0.001, Fig. 3). The pooled results revealed dramatically higher RFS in patients who received sorafenib. The data were analyzed using a random-effects model, and *I*^2^ for heterogeneity was 55%. Subgroup analysis was then conducted according to region, study design, analysis type (univariate or multivariate), and the presence of vascular invasion. As presented in Table 3, RFS was higher in Chinese patients in the fixed-effects model (pooled HR = 0.68, 95% CI = 0.57–0.81, *P* = 0.001). Patients with vascular invasion obtained a greater RFS benefit from sorafenib therapy according to the random-effects model, and *I*^2^ for heterogeneity was 52.5% (pooled HR = 0.51, 95% CI = 0.35–0.74, *P* < 0.001). Meanwhile, the pooled HRs were 0.53 (95% CI = 0.14–1.99, *P* = 0.35) for prospective studies and 0.7 (95% CI = 0.58–0.83, *P* < 0.001) for retrospective studies, and those for univariate and multivariate analyses were 0.91 (95% CI = 0.78–1.07, *P* = 0.274) and 0.51 (95% CI = 0.35–0.74, *P* < 0.001), respectively.

**Table 3:** HRs for RFS by subgroup

Subgroups	No. of studies	No. of patients	Random-effects model		Fixed-effects model		Heterogeneity	
			HR (95% CI)	*P*	HR (95% CI)	*P*	*I*^2^	*Ph*
**Regions**								
China	9	397	0.63 (0.49–0.81)	0.000	0.68 (0.57–0.81)	0.000	41.8%	0.089
Other countries	1	556	0.94 (0.78–1.13)	0.513	0.94 (0.78–1.13)	0.513	0%	0
**Study design**								
Prospective	2	570	0.53 (0.14–1.99)	0.350	0.91 (0.75–1.09)	0.292	82%	0.018
Retrospective	8	383	0.67 (0.53–0.85)	0.001	0.7 (0.58–0.83)	0.000	32.3%	0.170
**Root of HRs**								
Univariate	4	799	0.91 (0.78–1.07)	0.274	0.91 (0.78–1.07)	0.274	52.8%	0.060
Multivariate	6	154	0.51 (0.35–0.74)	0.000	0.62 (0.5–0.76)	0.000	0%	0.84
**Risk of factor (with vascular invasion or not)**								
Yes	6	240	0.51 (0.35–0.74)	0.000	0.62 (0.5–0.76)	0.000	52.4%	0.062
No	4	713	0.92 (0.78–1.07)	0.278	0.92 (0.78–1.07)	0.278	0%	0.855

*HR*, hazard ratio, *RFS* recurrence-free survival

**Fig. 4:**
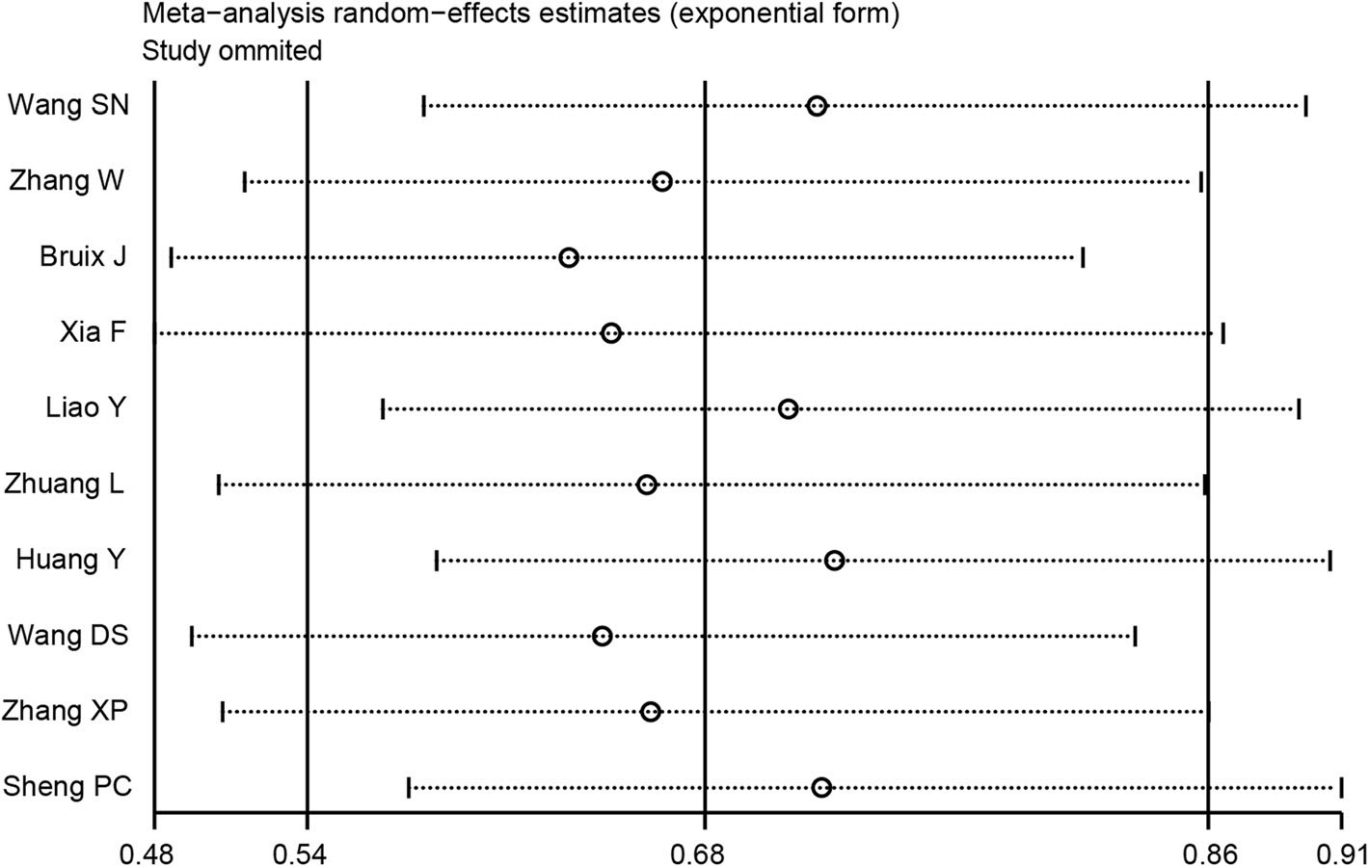
Sensitivity analysis of RFS. RFS, recurrence-free survival

Sensitivity analysis was performed using a “one-study removed” model. The results illustrated that the observed pooled HR for RFS was not significantly affected by removing any single study (Fig. 4). Publication bias for RFS was detected (Pr > |z| = 0.074 for Begg’s test and P > |t| = 0.005 for Egger’s test). The corrected pooled HR for RFS was not changed after using the trim and fill method to adjust for publication bias in the random-effects model (HR = 0.68, 95% CI = 0.54–0.86, *P* = 0.001).

#### Recurrence rate

**Fig. 5:**
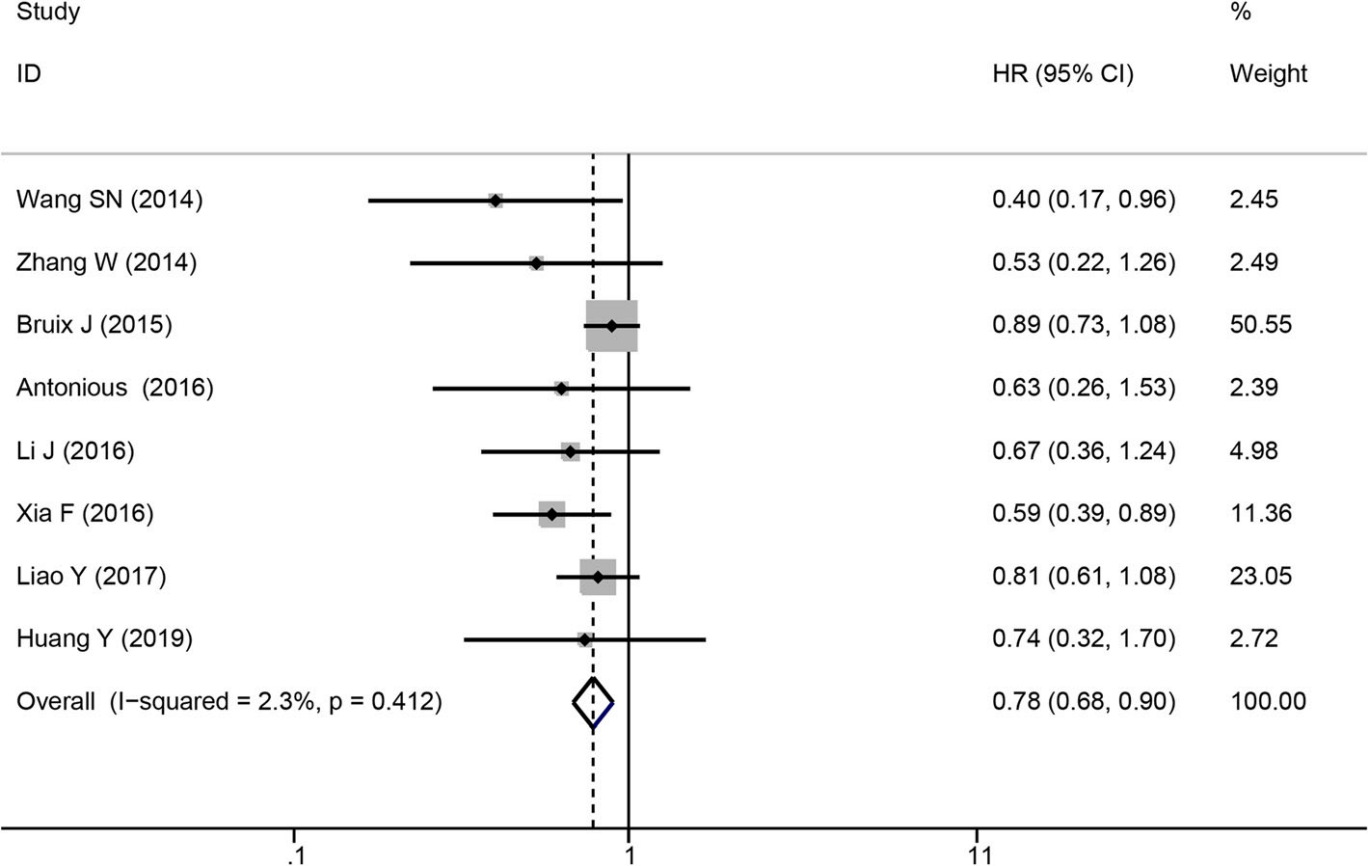
Forest plot of recurrence rate outcomes in patients who received sorafenib therapy for HCC after resection. HCC, hepatocellular carcinoma

Eight studies involving 1482 patients reported recurrence rates for sorafenib as adjuvant therapy in patients with resectable HCC. The combined data revealed that sorafenib treatment after resection was associated with a lower recurrence rate (pooled RR = 0.78, 95% CI = 0.68–0.90, *P* < 0.001), and no significant heterogeneity was found (*I*^2^ = 2.3%, *P* = 0.412, Fig. 5).

#### Adverse events

Seven studies provided adverse event data for adjuvant sorafenib treatment, although two studies only described the total incidence of adverse events without mentioning specific events. The most common sorafenib-related adverse reactions included hand-foot-skin reaction (HFSR), fatigue, alopecia, rash or desquamation, hypertension, anorexia, hematological events, transaminase elevation, and diarrhea (Table 4). HFSR was the most frequent event in four studies. Fatigue and diarrhea were also frequent events. According to the Common Terminology Criteria for Adverse Events, most adverse events were mild to moderate in severity, and grade 4 adverse reactions were rare (< 1%). Severe adverse events represent the major reason for sorafenib dose modification. All drug-related adverse events were resolved with treatment, and no adverse event-related deaths occurred.

**Table 4:** Adverse events occurring during adjuvant sorafenib therapy in seven comparative studies

Author	Sample size	Fatigue, N (%)		Hand-foot skin reaction, N (%)		Alopecia, N (%)		Rash or desquamation, N (%)		Hypertension, N (%)		Anorexia, N (%)		Hematological events, N (%)		Transaminase elevation, N (%)		Diarrhea, N (%)	
		All grades	Grade 3 or 4	All grade	Grade 3 or 4	All grade	Grade 3 or 4	All grade	Grade 3 or 4	All grade	Grade 3 or 4	All grade	Grade 3 or 4	All grade	Grade 3 or 4	All grade	Grade 3 or 4	All grade	Grade 3 or 4
Bruix J	556	73 (13)	9 (1)	389 (70)	154 (28)	179 (32)	–	91 (16)	4 (3)	108 (19)	24 (4)	33 (6)	2 (< 1)	32 (6)	9 (1)	100 (18)	56 (10)	215 (38)	34 (6)
Li J	12	NA	NA	11 (92)	NA	NA	NA	NA	NA	NA	10 (83)	NA	NA	NA	NA	NA	NA	10 (83.3)	NA
Chen BF	24	12 (50)	NA	10 (42)	NA	9 (38)	NA	7 (29)	NA	5 (21)	NA	14 (58)	NA	5 (21)	NA	NA	NA	7 (29)	NA
Zhuang L	27	3 (11.1)	0 (0)	16 (59.2)	2 (7.4)	3 (11.1)	0 (0)	5 (18.5)	0 (0)	1 (3.7)	0 (0)	3 (11.1)	0 (0)	NA	NA	NA	NA	5 (18.5)	1 (3.7)
Wang DS	98	NA	NA	3 (2.8)	NA	NA	NA	NA	NA	NA	NA	NA	NA	21 (21.4)	NA	20 (20.4)	NA	7 (6.5)	NA
Zhang W	32	Grade 3 adverse effects occurred in 6 patients with sorafenib treatment (18.8%)																	
Xia F	34	With only grades 1 and 2 drug-related adverse events (14 patients, 41.2% in this study)																	

*NA* not available

## Discussion

In this meta-analysis, we investigated 13 comparative studies, including 1 RCT and 12 retrospective studies, to explore the effects of adjuvant sorafenib therapy on survival and recurrence in patients with HCC who underwent radical resection. Our study found that adjuvant sorafenib can both improve RFS (HR = 0.68, 95% CI = 0.54–0.86, *P* = 0.001), reduce recurrence rates (HR = 0.78, 95% CI = 0.68–0.90, *P* < 0.001), and prolong OS (HR = 0.71, 95% CI = 0.59–0.86, *P* < 0.001).

The pooled outcomes of our meta-analysis illustrated that the use of sorafenib significantly prolonged OS. This result was inconsistent with that of a previous meta-analysis [25]. One of the reasons for the contradiction might be the differences in sample sizes and patient characteristics. The present study included more studies than the previous analysis, which is preferable in a meta-analysis setting. The previous meta-analysis only included six studies, and patients were divided into the control (reference) and sorafenib groups. However, each subgroup analysis included no more than four groups of data. Additionally, the former analysis did not conduct publication bias using funnel plots or Egger’s test, precluding definitive conclusions. Among the four additional studies, most patients underwent liver resection for HCC with histologically confirmed microvascular invasion or moderate-to-advanced HCC, which might explain the inconsistent results. However, there was publication bias concerning OS in this study, although when the trim and fill method was used to adjust for publication bias, the corrected pooled HR for OS did not change, which might be attributable to the number of original studies. When the study by Bruix was omitted [16], the sorafenib group still had better OS (HR = 0.52, 95% CI = 0.4–0.68, *P* < 0.001). No heterogeneity was observed among the studies (*I*^2^ = 0% *P* = 0.68), and no obvious publication bias was found (Pr > |z| = 0.175 for Begg’s test and P > |t| = 0.167 for Egger’s test).

Sorafenib, as a multi-target, multi-kinase inhibitor, both suppresses tumor proliferation by inhibiting serine/threonine kinases and blocking the RAF/MEK/ERK pathway and prevents tumor angiogenesis by inhibiting vascular endothelial growth factor receptors 1–3, platelet-derived growth factor receptor β, FMS-like tyrosine kinase 3, serine/threonine kinases (c-RAF and b-RAF), and epithelial growth factor receptor, thereby suppressing cancer growth and metastasis. Thus, the drug inhibits both tumor angiogenesis and tumor cell proliferation [12]. The availability of sorafenib for HCC treatment has been wildly acknowledged. In the phase III Sorafenib HCC Assessment Randomized Protocol (SHARP) study [2], sorafenib improved OS in patients with advanced, unresectable HCC by 3 months, and the safety and modest efficacy of sorafenib were validated in patients from the Asia-Pacific region [26]. A meta-analysis including 5125 advanced HCC patients revealed that there were significant improvements in overall survival (OS), overall response rate (ORR), and time to progression in patients who received TACE and sorafenib compared with the effects of TACE monotherapy [27]. Moreover, sorafenib can serve as a preoperative adjuvant therapy and downstage patients with large HCC, allowing further surgical resection [28]. Thus, the antiangiogenic, antiproliferative, and proapoptotic properties of sorafenib make it an ideal option after hepatectomy in theory. Animal studies revealed that sorafenib could control tumor growth and inhibit tumor recurrence after hepatectomy [29, 30]. However, clinical studies reported inconsistent results, mainly attributable to the high biological heterogeneity across HCC; thus, sorafenib might only have efficacy in certain patients or against particular activated signaling pathways. The Barcelona Clinic Liver Cancer staging and Child-Pugh cirrhosis classifications are the critical criteria for selecting patients with HCC who are suitable for sorafenib therapy [31]. In addition, an inflammatory microenvironment and circulating immune cells and cytokines play a significant role in the prognosis of HCC [32, 33]. To date, Hu et al. [32] used a systemic immune-inflammation index to forecast the prognosis of patients after curative resection. Other factors, including the viral status [34], adverse events attributable to sorafenib [35], fibroblast growth factor 3/fibroblast growth factor 4 amplification, angiogenic factors (angiopoietin-2 and vascular endothelial growth factor-A) [36, 37], have been reported to impair the efficacy of sorafenib. Several included studies reported that treatment prior to resection, tumor size, multiple tumors, intrahepatic metastasis, and vascular invasion were risk factors associated with the efficacy of sorafenib treatment.

Several limitations of this analysis must be considered. First, the inclusion of studies with different study designs, including retrospective cohort studies, retrospective case-control studies, and an RCT, might have affected the outcome of the analysis. Hence, RCTs with larger patient populations are needed to confirm the present outcomes. Second, only a small number of studies were included (13 articles), and included studies were constrained to those published in the English language, resulting in selection biases. Moreover, publication bias was observed for OS, and high heterogeneity was observed for RFS, which might affect the interpretation of the results of this meta-analysis. Thus, definitive conclusions concerning the efficacy of sorafenib in patients with HCC after resection could not be drawn.

## Conclusions

In summary, this meta-analysis demonstrated that sorafenib as an adjuvant treatment in patients who underwent resection for HCC could prolong OS, improve RFS, and reduce recurrence rates without intolerable adverse effects. Sorafenib might be an effective option for preventing HCC recurrence after resection. However, the results of this meta-analysis need to be interpreted with caution because sorafenib might only be effective in patients with certain risk factors or those with particular activated signaling pathways. In the future, more well-designed, large-scale studies are needed to confirm these findings.

## Data Availability

The data and materials were obtained and analyzed from the current study or acquired from the corresponding author on reasonable request.

## Abbreviations

HCC: Hepatocellular carcinoma
OS: Overall survival
RFS: Recurrence-free survival
HR: Hazard ratio
RR: Pooled risk ratio
RES: Surgical resection
RFA: Radiofrequency ablation
AEs: Adverse events
HFSR: Hand-foot skin reaction
